# Frequency and Associated Factors for Care Giving among Elderly Patients Visiting a Teaching Hospital in Karachi, Pakistan

**DOI:** 10.1371/journal.pone.0025873

**Published:** 2011-11-04

**Authors:** Waris Qidwai, Mohammad Uzair Abdul Rauf, Seema Sakina, Ayesha Hamid, Sidra Ishaque, Tabinda Ashfaq

**Affiliations:** 1 Department of Family Medicine, Aga Khan University, Karachi, Pakistan; 2 Dow University, Karachi, Pakistan; 3 Ziauddin University, Karachi, Pakistan; Universidad Peruana Cayetano Heredia, Peru

## Abstract

**Objective:**

To study frequency and associated factors for care giving among elderly patients visiting a teaching hospital in Karachi, Pakistan.

**Methodology:**

A cross sectional questionnaire-based study was conducted at the Community Health Centre (CHC), Aga Khan University Hospital (AKUH) Karachi, Pakistan from September to November 2009. All individuals, visiting the CHC and aged 65 years or above were interviewed after taking written informed consent.

**Results:**

A total of 400 elderly completed the interview. Majority were females, 65–69 years age, More than half of the individuals ie: 227 (85%) had received Care Giver experience for assistance and among these 195(72%) had care provided by an immediate family member. A large proportion of them stated that their Care Givers managed to provide less than four hours in a day for care giving. Around 37% showed substantial improvement in their relationship with the care givers. About 70% of the respondents stated that the care provided by the Care Giver improved their quality of life.

**Conclusion:**

Elderly care is provided by majority of the family members resulting in increased satisfaction level, however small number still not satisfied due to unfulfilled need of these older people. This demands that efforts should be made to strengthen the family support by increasing awareness regarding elderly care and arranging support system by the government.

## Introduction

Elderly population particularly aged 65 years and more contributes to 380 million people globally and by the year 2020, this population is projected to increase to more than 690 million [1], [2]. Pakistan is the sixth most populous country in theworld with an estimated population of 166 million incorporating nearly 7 million of elderly population [3].

This older population is vulnerable to various disabilities as a consequence of stroke, dementia, heart diseases and trauma [4], [5]. This demonstrates the ever increasing need for Care Givers in the community to look after geriatric population with disabilities. Care giving can occur at any point in the life-course, for the older population suffering from chronic illnesses or disabilities, which result in loss of independent functioning. Services rendered by the Care Givers can ensure better quality of life for these disabled persons.

According to Center for Disease Control (CDC), about 80% of older Americans are living with at least one chronic condition, and 50% with two [6]. Elderly people usually encounter dietary, medical or physical and social problems. They also encounter unique nutritional challenges as a consequence of chronic medical conditions, such as Osteoporosis,Arthritis, Depression and Diabetes Mellitus etc [7]. Majority of elderly people live alone and thus have less nutritious diets in comparison to those living with a family [7].

Care Givers can either be formal (paid volunteers associated with a service system) or informal which includes family, friends, neighbors or trust members who provides unpaid care out of love, respect, obligation or friendship to a disabled person [8]. Family members among the Informal Care Givers are of paramount importance in providing care, especially in Pakistani culture and society where family ties and bonding is a norm. Family members can very well assist in activities of daily living (ADL), such as grooming, walking,eating/feeding etc and also provide emotional support to these dependent individuals [9]. The economic value of the informal care giving by studies have shown it to be worth hundreds of billions of dollars [10].

In accordance with accepted social and cultural norms in Pakistan, people prefer to live in an extended family system as it is cost effective and has many other advantages including availability of support for the elderly by the family members [11].

There is serious scarcity of formal Care Givers and also non availability of Trusts and social projects to provide care to the elderly people in Pakistan, we designed this study to assess the problems faced by the people requiring support from Care Givers and the barriers faced by the care givers in providing support., so that proper intervention techniques could be devised to raise the quality of life of the elderly and to reduce the negative outcomes of care giving practices.

## Materials and Methods

This was a cross sectional survey conducted at the Community Health Centre (CHC), Aga Khan University Hospital (AKUH) located at Karachi from September 2009 to November 2010. AKUH is a tertiary level teaching facility in the private sector and caters to the medical needs of a large majority of patients coming from all over Karachi as well as from other parts of Pakistan. CHCalong with family medicine clinics also provides specialist services to a number of patients thus enabling a diverse assortment of patients from all backgrounds to be encountered.A total of 423 people who were 65 years or older presenting to CHC for consultation were approached out of which 400 agreed to participate in the study through non probability convenient sampling.

### Sample size

Sample size was calculated through WHO sample size calculator by using following assumptions: since we do not have any prevalence data for the Frequency and associated factors for care giving among elderly patients visiting a teaching Hospital in Karachi, Pakistanhence a prevalence of 50% was assumed to observe a difference, with bound of error of 5% and level of significance of 5%, the estimated sample size was 385.After addition of 10% for non-response the final sample size was approximately 423 study participants.

### Questionnaire

#### Development

The initial questionnaire was designedbased on the prior experience of investigators, input fromcolleagues, peers as well as patients.The proforma was then pre-tested on 25 respondents and no changes were deemednecessary to be made in the questionnaire based on this pretesting.

#### Linguistic validation of questionnaire

The questionnaire was initially prepared in English; then translated into Urdu, the national language of Pakistan, for the convenience of the respondents. The questionnaire was also back-translated into English by two non-medical personnel to check for any paraphrasing errors.

#### Sections

The questionnaire was divided into four sections and included questions related to Socio demographic factors, needs of elderly people and their choices for Care Givers, perception of elderly people regarding their Care Givers and the impact of Care Giving on their life. And lastly characteristics of care giving among respondents based on gender.

### Statistical Analysis

Data was entered using Epi-data version 3.1 and analyzed through SPSS version 15. Frequencies were calculated for categorical variables (Gender), Mean and standard deviation for age. Cross tabulation was done and chi-square test was applied to compare different factors related to care giving on the basis of gender and *p*-value of <0.05 was considered significant.

### Ethical Consideration

Written informed consent was obtained from the subjects after explaining the study objectives. The subjects were free to withdraw at any time without giving any reason.Strict confidentiality was maintained throughout the process of data collection, entry and analysis. All efforts were made in this study to fulfill the ethical considerations in accordance with the ‘Ethical principles for medical research involving human subjects’ of Helsinki Declaration [12]. The procedures were reviewed and approved by the Ethics Committee of the Aga Khan University Hospital.

## Results

A total of 423 people were approached out of which 400 agreed to participate in the survey.The overall response rate was found to be 91%.Among these respondents, 165(41%) were male and 235 (59%) female with a mean age of 69±5.95 years.

Table 1 shows the socio demographic characteristics of study population. Majority of the respondents were married (98%) and had three or more children (77%), providing opportunities of care giving by children. Regarding profession majority of the respondents 279 (70%) were retired. The financial status of nearly half of the respondents (47%) was found to be between Rs. 10, 000 to 50,000 per month. As far education status was concerned 113(28%) of the participants were graduate and higher.

**Table 1:** Demographic profile of respondents.

Respondents		Frequency (N = 400)	%
**1.Gender**			
1	Males	165	41
2	Females	235	59
**2. Age distribution(yrs)**			
1	65–69	243	61
2	70–74	65	16
3	75–79	37	9
4	80–84	55	14
**3.Marital status**			
1	Married/Ever married	393	98
2	Single/unmarried	7	2
**4. Children**			
1	No child	24	6
2	One child	25	6
3	Two children	45	11
4	Three or more children	306	77
**5.Occupation**			
1	Employed	121	30
2	Unemployed	279	70
6.Income (in rupees*)			
1	<5000	46	12
2	5000–10000	93	23
3	>10000–50000	188	47
4	>50000–100000	45	11
5	>100000	28	7
**7.Level of education**			
1	Illiterate	120	30
2	Primary	43	11
3	Intermediate	124	31
4	Graduation/post-graduation	113	28

Table 2 shows the characteristics of care giving and factors associated with it. More than half of the respondents 268 (67%) felt the need of service by a Care Giver and around 85% had access to care givers whereas only 15% did not have any care givers to fulfill their needs.

**Table 2:** Characteristics of Care Giving and factors associated with it.

Respondents	Frequency	%
**1.Needs care giver service (n = 400)**		
Yes	268	67
No	132	33
**2.Care givers present for your needs (n = 268)**		
Yes	227	85
No	41	15
3.Relationship with the care giver*		
Family members	195	-
Relative	25	-
Neighbor	6	-
House maid	40	-
Health care providers	5	-
**4.Impact on relationship with the care giver after receiving care (n = 227)**		
Improved	85	37
Detoriated	8	4
No change	134	59
**5.Did you get any Improvement in general well being (n = 227)**		
Yes	158	70
No	69	30
5.Improvement observed in the general well being after care provided: **		
Financially	33	-
Medically	116	-
Physically	74	-
Emotional	51	-
Social	39	-
Spiritual	5	-
**6.Did Your care givers faced any barriers in providing care (n = 227)**		
Yes	98	43
No	129	57
**7.Barriers which prevent care givers to provide care (n = 98)**		
Financial	29	30
Lack of time	29	30
Health reasons	6	6
Job responsibilities	22	22
Home responsibilities	12	12

(* multiple responses so n cannot be 227 and there percentage cannot be computed).

(** multiple responses so n cannot be 158there percentage cannot be computed).

On enquiring about the relationship of the care givers with the participants around three fourth of the care givers (72%) were their immediate family members whereas only (15%) were house Maids and (9%) their relatives. Relationship of the elderly with the care givers have improved in only (37%) cases while (59%) did not felt any change at all. Impact of care giving to respondents showed improvement as mentioned by majority of the respondents and .on specifying improvements areas majority responded for medical problem (37%) followed by improvement in financial (10%), social (12%) and emotional well being (16%).Problems in providing service was faced by (43%) of the care providers as mentioned by care recipients. Most common reasons responsible for slow down of taking care of elderly people/relatives included financial difficulty (30%) lack of time(30%) followed by job responsibility(22%),home responsibilities(10%) and, health reasons(6%)respectively.

Table 3 shows characteristics regarding care givers based on gender. Preference was given to care givers from family members by more than three forth of people (86%) majority from females with (p-value = 0.048)and most of the respondents (96%) including both the gender were satisfied with the care they received from the care providers (p-value = 0.001).Time given to the respondents by more than half of the care givers (52%) was less than four hours daily as expressed by both the gender(p-value = 0.091),on the other hand small number of care taker (21%) spent 8 hrs//day. On inquiring about duration of assistance from the care takers female required care for more than 3 yrs whereas male required helped for 1–3 yrs ((p-value = 0.001).

**Table 3:** Characteristics of Care Giving among respondents based on Gender.

S.No	Variables	Male	Female	p-value
**Preference of Family member as a care taker**				
1	Yes	69	126	0.055
2	No	17	15	
**Time given by a care taker in a day**				
1	Less than 4 hrs	45	72	0.091
2	4–8 hrs	12	35	
3	8–16 hrs	10	17	
4	16–24 hrs	19	17	
**Duration of dependence on care giver**				
1	Less than 6 months	22	17	0.001
2	6 months–6 yrs	12	15	
3	1–3 yrs	27	32	
4	More than 3 yrs	25	77	
**Preference of elderly home care**				
1	Yes	25	16	0.001
2	No	61	125	
**Satisfies with care received from home care giver**				
1	Yes	78	139	0.005
2	No	8	2	

It is important to note that 290 (73%) respondents reported they provided care in their past life and among these 66% of them acted as a Care Giver for their family members. Regarding duration of care giving 54% of the participants recalled that they provided care for more than 3 years. About 68% of them accepted that they faced difficulty in providing care with majority having financial difficulty and lack of time as the main barrier.

**Figure 1:**
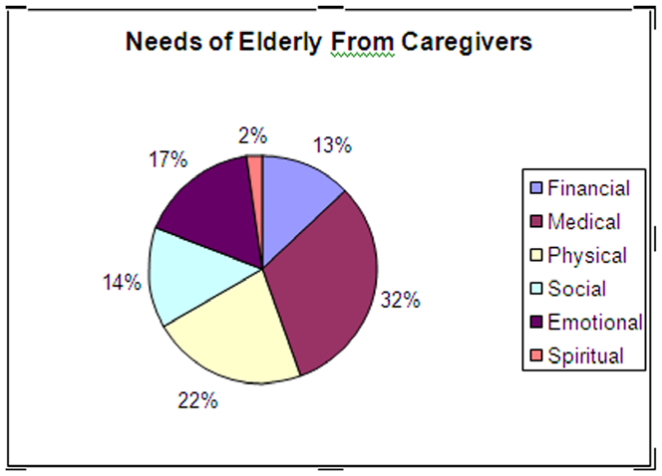
Needs of Elderly for Caregivers.

Figure 1 shows needs required by elderly population from their care givers. Major areas of the requirement for the care by care givers were reported to be Medical (31.5%), Physical (22.4%) and Emotional (17.4%). Financial support was needed by 89 (12.7%) respondents andsocial and spiritual needs were reported by 13.8% and 2% of the elderly population respectively.

## Discussion

This study assessed frequency and associated factors of care giving among elderly population visiting a teaching hospital in Karachi. Numerous studies have been conducted worldwide but to the best of author's knowledge very limited data exist regarding trends of care giving in Pakistan.

In this study majority of the participants were females probably females required more assistance of care givers in comparison to males and they have higher life expectancy as compared to males these results are consistent with studies in other parts of the world [13], [14]. whereas another study done in Pakistan shows males as main seeker for health care [15]. Most of these participants are retired similar to a study done in Pakistan [15]. Probably because retirement age in Pakistan is 60 yrs and very limited support is provided to these people from the government thus making them financially dependent on their family members.

It was seen that majority of the elderly needed care givers for medical and physical reasons as large number of population in this age group suffers from multiple Co-morbidities [16] [5]. Only small numbers require emotional assistance and this can be explained on the basis of existence of fast life in urban population with less time available for emotional interactions and exchanges. The traditional trends of extended family model being replaced by nuclear family model are also resulting in lack of emotional support for the elderly.

Similarly financial needs were also reported by only small number of respondents probably because they belonged to educated and financially better placed group of individuals. It also is explained on the basis of support being available to those living in extended family model.

Social needs were stated by small number of respondents because of their limited mobility as a consequence of multiple co-morbidities and physical limitations. A need exists to promote Senior Citizen Clubs and societies that may provide opportunities for socializing among elderly population and social interactions and exchanges.

A majority of respondents reported immediate family members specially females as care providers similar to another study done in Thailand [17], [18].

This will lead to a serious issue in future with the substitute of traditional extended family system with nuclear family system. On the other hand it was interesting to note that small numbers of respondents were using House Maid as care givers.

Time provided by the care givers by half of the elderly population was less than four hours per day and only a small number were able to give up to eight hours of care giving which is required for minimum needs. Another aspect was that there were more than one Care Giver for a given patient and the added supported may be able to satisfy most of the patient requirements this is consistent with a study done in Spain which showed care givers spent around 14 hrs/day with more than one care givers [19].

It is also notified that a significant respondents required assistance from Care Giver for more than three years similar results of study done in Pakistan on care giving of cancer patients [20].This highlights the long term need for care giving in these patients thus requiring strategies to ensure availability of such services on an ongoing basis.

Relationship between patient and care provider has an important role to play in care giving as evident by a prior studies [19], [21]. In this study half of the respondents reported no change in their relationship with care giver after need for care giving arose whereas more than one third respondents reported an improvement in relationship probably care givers came closer to and spend more time with the elderly and resulted in better understanding and relationship. Care giving has become an important challenge for the family members as presence of traditional family system favours majority of care giving provision by the family memberstherefore compromising their privacy, increasing burden financially and mentally and ultimately leads to increasing incidents of conflicts among them and elderly people highlighting the importance of need of proper planning for the care giving.

It was heartening to see that around one third of the respondents reported improvement after care giving. Major areas of Improvements were medical, physical, social and financial well being [18], [22].

On further questioning one third of the respondents reported unfulfilled needs despite provision of care giving. Financial need was the most commonly reported unfulfilled need. These results are in contrary to findings of a study done in Japan on females with or without dementia and showed majority of the females without dementia were satisfied with their daily life and economic status [22]. This highlights the need for pension plans and other insurance plans that can provide financial support in the retired population.

Nearly two third of the respondents reported that their needs were fulfilled by care giving. Majority of the people had an impression that the care provided by the care giver improved their quality of life [22].This is very important because quality of life in the main focus of providing care to the elderly population.

Majority of the respondents reported they provided care in their past life and more than half of them acted as a Care Giver for their family members. This shows that Care Giving is an accepted practice in Pakistani society. Half of these individuals recalled that they provided care for more than 3 years, again emphasizing long term and ongoing nature of care giving in the elderly.

On further enquiring about difficulties which they faced while providing care more than half of the respondents faced difficulty in providing care with a majority having financial difficulty and lack of time as the main barriers these results are similar to a study done in Canada and Spain [19], [23]. Exploration into barriers to provision of care giving can help develop strategies that can remove impediments and help provide long term care to those who need it. All the individuals who provided care in their past life stated that being a Care Giver gave them internal satisfaction and feeling of well being. This point is important in promoting care giving in the society.

### Conclusion

This study highlights the issues and problems encountered by elderly people and their care givers and also clearly demonstrate the level of satisfaction regarding their care. Elderly care is provided by majority of the family members resulting in increased satisfaction level, however small number still not satisfied due to unfulfilled need of these older people as a consequence of hindrance faced by the family members in the form of financial, mental and psychosocial issues. Lack of awareness among family members for elderly care is also an important area of concern. Pakistan lack social services or study respite centers or any support system for the elderly. This situation demands efforts to strengthen the family support by increasing awareness regarding elderly care and arranging support system by the government for these older population.

### Limitations

This study was done on a selected population of elderly in a tertiary hospital which caters mostly educated people with good financial background so it cannot be generalized. However Community Health Centre caters people from all socioeconomic groups this fact helped us in offsetting this particular limitation of study.

## References

[1] World Health Organization The World Health Report 1997, 50 facts[online] 1997.. http://www.who.int/whr/1997/media_centre/50facts/en/index.html.

[2] World Health Organization (1999). Ageing-exploding the myths.

[3] Population Reference Bureau (2006). The 2006 World Health Data Sheet[online].

[4] National Institute on Aging (2010). Hearts and arteries: Health for older adults. [Online].. http://www.enotalone.com/article/10799.html.

[5] Saleem T, Khalid U, Qidwai W (2009). Geriatric patients' expectations of their physicians: findings from a tertiary care hospital in Pakistan.. BMC Health Serv Res.

[6] Centers for Disease Control and Prevention and the Merck Company Foundation (CDC & Merck) (2007). The state of aging and health in America 2007.

[7] Nutrition and Well-Being A to Z Aging and Nutrition [Online] 2010.. http://www.faqs.org/nutrition/A-Ap/Aging-and-Nutrition.html.

[8] Cummings SM, Kropf NP (2009). Formal and informal support for older adults with severe mental illness.. Aging Ment Health.

[9] Maas ML, Reed D, Park M, Specht JP, Schutte D (2004). Outcomes of family involvement in care intervention for caregivers of individuals with dementia.. Nursing Research.

[10] Arno PS, Levine C, Memmott MM (1999). The economic value of informal care giving.. Health Aff.

[11] Itrat A, Taqui AM, Qazi F, Qidwai W (2007). Family systems: perceptions of elderly patients and their attendants presenting at a university hospital in Karachi, Pakistan.. J Pak Med Assoc.

[12] World Medical Association Declaration of Helsinki http://www.wma.net/en/30publications/10policies/b3/index.html.

[13] Brody EM (1981). Women in the middle and family help to older people.. Gerontologist.

[14] Gibson MJ, Nursberg C (1984). Family support patterns, policies and programmes.. Innovation ageing programmes abroad. Implications for the United States.

[15] Zafar SN, Ganatra HA, Tehseen S, Qidwai W (2006). Health and needs assessment of geriatric patients: Results of a survey at a teaching hospital in Karachi.. J Pak Med Assoc.

[16] World Health Organization (1999). Ageing - Exploding the Myths.

[17] Laubunjong C, Phlainoi N, Graisurapong S, Kongsuriyanavin W (2008). The pattern of care giving to the elderly by their families in rural communities of Suratthani Province.. ABAC Jour.

[18] Wang YC, Chung MH, Lai KL, Chou CC, Kao S (2004). Preferences of the elderly and their primary family caregivers in the arrangement of long-term care.. J Formos Med Assoc.

[19] LÃ pez J, LÃ pez-Arrieta J, Crespo M (2005). Factors associated with the positive impact of caring for elderly and dependent relatives.. Arch Gerontol Geriatr.

[20] Yousafzai AW, Bhutto N, Ahmer S, Siddiqi MN, Salamat S (2008). Care Givers' stress of cancer patients in a tertiary care hospital.. JPMI.

[21] Gupta R (2000). A path model of elder caregiver burden in Indian/Pakistani families in the United States.. Int J Aging Hum Dev.

[22] Onishi C, Yuasa K, Sei ML, Ewis AA, Nakano (2010). Determinants of life satisfaction among Japanese elderly women attending health care and welfare service facilities.. The Jour Med Invest.

[23] Grunfeld E, Glossop R, McDowell I, Danbrook C (1997). Caring for elderly people at home: the consequences to caregiver's.. Can Med Assoc J.

